# Assessment of Knowledge Retention in Military Personnel After Training Courses in Sieve Triage Using Different Simulated Scenarios

**DOI:** 10.7759/cureus.23484

**Published:** 2022-03-25

**Authors:** Omar Ghazanfar, Saleh Fares, Ahmed H Mubarak, Ives Hubloue

**Affiliations:** 1 Emergency Department, Zayed Military Hospital, Abu Dhabi, ARE; 2 Emergency Medicine Department, Zayed Military Hospital, Abu Dhabi, ARE; 3 Emergency and Disaster Medicine, Emergency Medicine Department - Research Group on Emergency and Disaster Medicine, Universitair Ziekenhuis Brussel, Vrije Universiteit Brussel, Brussels, BEL

**Keywords:** skills and simulation training, course delivery, knowledge retention, mass casualty, keywords: sieve triage

## Abstract

Introduction

In the course of their pre-deployment training, military students in the United Arab Emirates (UAE) are instructed on sieve triage, which is used on the battlefield. The objective of this study is to test the retention of knowledge immediately after delivery of the triage course, at Day 30 and Day 60 in military personnel with no previous sieve-triage knowledge and with an undifferentiated professional background.

Method

Data were collected using a questionnaire based on a survey toolkit designed by the University of Washington Public Health and distributed randomly amongst military personnel after delivery of sieve triage training. The students were randomly selected in consecutive cohorts over a six-week period.

Results

A total of 456 participants were included in the study. Most of the participants were soldiers (80%); other professions included were officers (9%), nurses (1%), paramedics (1%), and others (9%). The overall mean score for the cohort was 96.81 at Day 0; 87.37 at Day 30; and 76.1 at Day 60. The mean scores depict a decreasing trend for the combined as well as the individual cohorts with the highest mean score at Day 0 and the lowest at Day 60. The mean scores reduced significantly at Day 30 (MD: -9.43; 95% CI: -10.73 to -8.14) and at Day 60 (MD: -20.71; 95% CI: -22.01 to -19.42) compared to Day 0. The mean difference remained significant at Day 30 (MD: -9.42; 95% CI: -10.7 to -8.14) and Day 60 (MD: -20.69; 95% CI: -21.97 to -19.41) compared to Day 0 when adjusted for age and profession.

Conclusion

Knowledge retention from the delivery of sieve triage training among UAE military personnel decreased after 60 days. Therefore, there is a need for regular and periodic refresher courses and training, especially for topics that are not applied regularly.

## Introduction

Triage is derived from the French word “trier,” meaning “to sort or sieve.” In daily practice, this process aims at prioritizing patients presenting to the emergency department based on the severity of their illness [1]. In these circumstances [2], it is often referred to as “doing the best for everyone” [3-4].

Originally, the process of triage was developed for use in military conflicts [2]. Besides the process of sorting patients in order of priority for treatment, the need for an evacuation from the battlefield is taken into account in the triage of wounded soldiers. Based on this principle, triage is equally applicable to “civilian” disaster settings and is therefore referred to as “doing the best for the most” [5].

Correct identification of those casualties that need the most urgent intervention, as well as quickly and safely identifying those who can wait longer for treatment is the basis for accurate triage. Correct performed triage also allows the identification of casualties who are so severely injured that they will not survive. In these cases, their treatment will tie up the (often scarce) available resources that may be best used for other victims [1-2]. 

There are a lot of triage methodologies that have been developed but evidence-based research into the efficacy of these various tools is difficult [1] Actually, there is no gold standard against which different triage tools can be measured. It has also not been proved that triage is better performed by doctors compared to nurses or other healthcare workers. On the contrary, repetitive training of a specific triage methodology is the only way to keep its accuracy at the expected level [6-8].

In military settings, the first triage is done on the battlefield [2]. A second triage is often performed at the Casualty Collecting Point or the Advanced Medical Post. The first triage should be fast, reliable, reproducible, easy to use, and easy to teach. Based on these characteristics several organizations (ambulance services, search and rescue teams) often apply the sieve triage methodology on casualties at the incident site in non-medical locations. In military conditions, this is referring to the battlefield [2].

The sieve triage methodology identifies immediately life-threatening problems based on the C < A B C > system and correctly prioritizes the patients for immediate treatment (see the algorithm in the Appendices) [2].

Sieve triage categorizes patients as:

P1 or red tags (immediate): are used to label those casualties that cannot survive without immediate care but have a chance of survival.

P2 or yellow tags (observation): are used for casualties that require observation and subsequent re-triage. The clinical condition at the current juncture is deemed stable and not in immediate danger of death. These victims will eventually require definitive care in hospital care and would be treated immediately under normal circumstances.

P3 or green tags (wait): are reserved for the 'walking wounded' who will require medical care at some point after more critical injuries have been treated.

P4 or P1E (expectant): is used for those whose injuries are so extensive that these would be considered un-survivable injuries given existing resources. This is used under the authorization of the Medical Incident Officer who has the responsibility to match these patients' injuries with other casualties and the available resources available to the hospitals.

Sieve triage is applicable for adult victims, but similar principles also apply to pediatric patients. In practice “pediatric triage tapes” are available, which groups children by length, weight, and age and provides normal physiological values for respiratory rate and pulse in each of the groups to properly carry out the triage process [2].

The UAE Military Medical Education directorate is tasked to deliver a number of medical courses to non-medical personnel pre-deployment to conflict zones to optimize the care of battlefield casualties. This includes the teaching of SIEVE triage to soldiers, officers, and other healthcare workers active at the front line in conflict zones.

The aim of this study was: (1) to assess the level of competence of military personnel in the use of sieve triage using a pre and post-course questionnaire and (2) to assess the knowledge retention at Day 0, Day 30, and Day 60 post-delivery of the course.

## Materials and methods

Between the 6th of January and 15th of February 2019, sieve triage training was delivered to six consecutive cohorts of students randomly assigned and with no pre-selection. The cohort was un-differentiated and included soldiers, officers, and nurses. Each cohort had no previous training with sieve triage. The training material was identical in all six cohorts.

An identical end-of-course exam was designed with 11 clinical scenarios. All cohorts were re-examined at Day 30 and Day 60 with 11 further questions (see the Appendices with examples of questions), each of which was different from Day 0 but identical to all cohorts at Day 30 and then separate at Day 60 but identical to all cohorts at Day 60. The same examiner pool was used for all questions and no further course or training material was delivered between Day 0 and Day 60. A 100% re-examination at Day 30/Day 60 was ensured.

The data were collected using a questionnaire based on a survey toolkit designed by the University of Washington Public Health and distributed randomly amongst Military personnel after delivery of the training [6]. Data were collected and analyzed with traditional descriptive statistical tools to reach a final conclusion.

Data were collected on participant scores, age group, and profession. Mean scores were calculated for the individual cohorts as well as the overall cohort at Day 0, Day 30, and Day 60. The difference between the mean scores between Day 0 and Day 30 and Day 0 and Day 60 was calculated and compared as mean difference (MD). The overall mean scores were compared across the age groups and professions. We also assessed whether the MD (from Day 0 to Day 60) differed across the various age groups and professions. We report the findings as mean scores and MD in the scores with corresponding 95% confidence intervals (CIs).

## Results

A total of 456 participants (Cohort 1=66; Cohort 2=82, Cohort 3=71; Cohort 4=72; Cohort 5=81, Cohort 6=84) were included in the study. Participants ages ranged from age group: 18-24; 25-34; 35-44; and 45-54 years. Most of the participants were military soldiers (80%); other professions included were officers (9%), nurses (1%), paramedics (1%), and others (9%).

**Figure 1:**
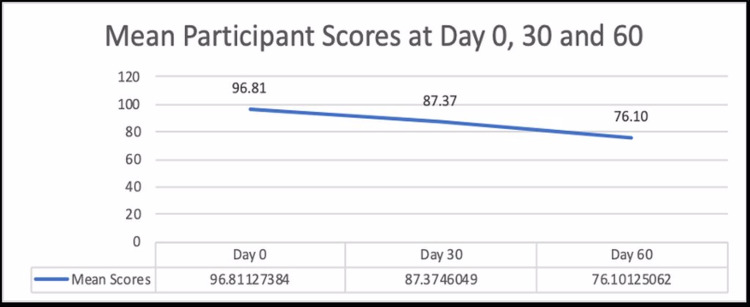
Mean participant scores at Day 0, Day 30, and Day 60 for the overall cohort

**Figure 2:**
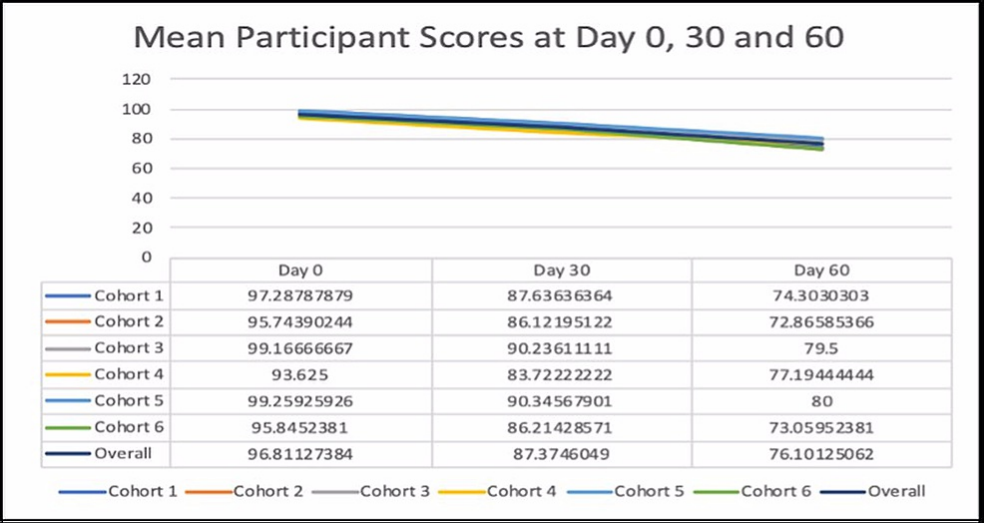
Mean participant scores at Day 0, Day 30, and Day 60 for the individual and combined cohorts

The overall mean scores for the cohort were 96.81 at Day 0; 87.37 at Day 30; and 76.1 at Day 60 and are presented in Figure 1. Figure 2 depicts the individual cohort mean scores at Day 0, Day 30, and Day 60. The mean scores depict a decreasing trend for the combined as well as the individual cohorts with the highest mean score at Day 0 and the lowest at Day 60.

**Figure 3:**
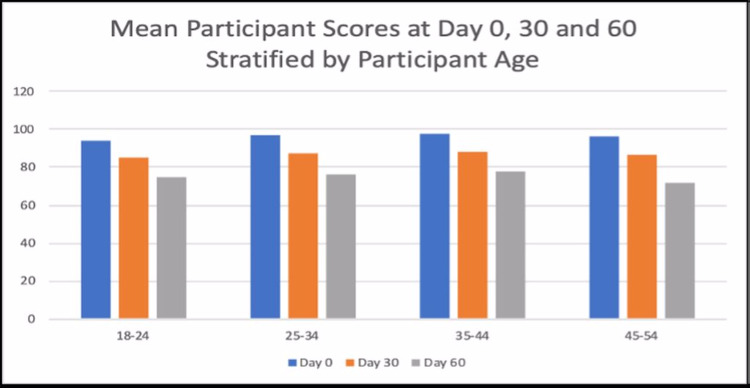
Mean participant score at Days 0, 30, and 60 stratified by participant age

**Figure 4:**
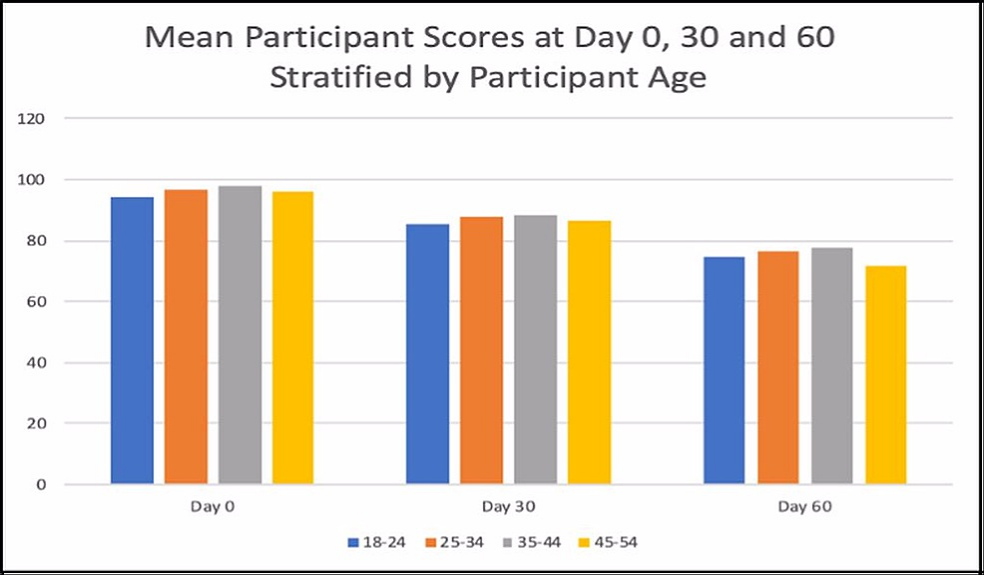
Mean participant score at Days 0, 30, and 60 stratified by participant age

Figure 3 and Figure 4 are reflecting the mean scores of all the participants stratified by age. In all the age groups, the mean scores appear to be declining from Day 0 to Day 60.

**Figure 5:**
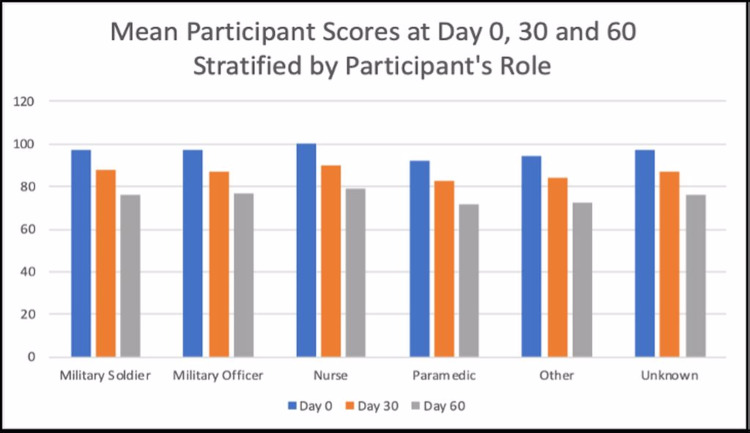
Mean participant score at Days 0, 30, and 60 stratified by participant age

**Figure 6:**
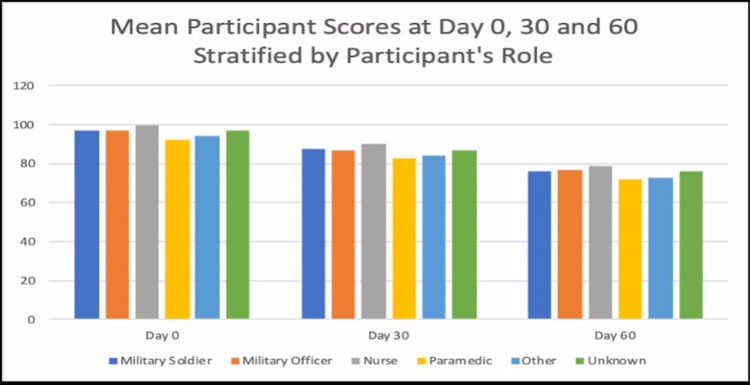
Mean participant score at Days 0, 30, and 60 stratified by participant age

Figure 5 and Figure 6 are showing the mean scores of all the participants stratified by their role/profession. Across all the professional roles, the mean scores appear to be declining from Day 0 to Day 60.

Regression analysis for the overall mean scores at Days 0, 30, and 60 was conducted. The regression analysis suggested that at the univariate level, the mean scores reduced significantly at Day 30 (MD: -9.43; 95% CI: -10.73 to -8.14) and at Day 60 (MD: -20.71; 95% CI: -22.01 to -19.42) compared to Day 0. The mean difference remained significant at Day 30 (MD: -9.42; 95% CI: -10.7 to -8.14) and Day 60 (MD: -20.69; 95% CI: -21.97 to -19.41) compared to Day 0 when adjusted for age and profession.

When the overall mean scores were compared across the age groups, the mean scores were significantly higher for the 25-34 age group (MD: 2.15; 95% CI: 0.61 to 3.69) and 35-44 age groups (MD: 3.05; 95% CI: 1.49 to 4.61) compared to the 18-24 age group, both at the univariate and the multivariate levels. The overall mean scores did not significantly differ for the 45-54 age group (MD: -0.24; 95% CI: -2.43 to 1.96) compared to the 18-24 age group both at the univariate and multivariate levels.

When the overall mean scores were compared across the professions, the mean scores did not statistically differ for any of the professions when compared to the military officer at the univariate level. At the multivariate level, the overall mean scores were significantly lower for the ‘other’ profession compared to the military officer (MD: -3.9; 95% CI: -6.6 to -1.21). There was no difference in the overall mean scores for any of the other professions compared to military officers at the multivariate level. Table 1 reports the unadjusted and adjusted estimates for the overall mean scores.

**Table 1:** Mean scores with unadjusted and adjusted covariates

Mean scores with unadjusted and adjusted covariates							
	Unadjusted				Adjusted		
	Mean	Mean Difference	95% CI	p-value	Mean Difference	95% CI	p-value
Day of Training							
Initial Day	96.81		Ref.			Ref.	
Day 30	87.37	-9.43	-10.73, -8.14	< 0.0001*	-9.42	-10.70, -8.14	< 0.0001^*^
Day 60	76.10	-20.71	-22.01, -19.42	< 0.0001*	-20.69	-21.97, -19.41	< 0.0001^*^
Age Group							
18-24	84.81		Ref.			Ref.	
25-34	87.06	2.26	0.24, 4.27	0.028*	2.15	0.61, 3.69	0.006*
35-44	87.83	3.02	0.98, 5.06	0.004*	3.05	1.49, 4.61	< 0.0001*
45-54	84.83	0.02	-2.88, 2.92	0.987	-0.24	-2.43, 1.96	0.833
Profession							
Military Officer	87.02		Ref.			Ref.	
Emergency Nurse	89.33	2.31	-6.50, 11.12	0.607	1.79	-4.87, 8.45	0.598
Military Soldier	87.02	0.001	-2.38, 2.38	0.999	0.08	-1.72, 1.89	0.928
Other	83.46	-3.56	-7.11, -0.02	0.049	-3.9	-6.60, -1.21	0.005*
Paramedic	82.40	-4.62	-11.59, 2.35	0.194	-4.95	-10.24, 0.33	0.066
Practical Nurse	90.33	3.31	-11.61, 18.23	0.664	3.09	-8.19, 14.37	0.591
Professional Role unknown	90.00	2.98	-2.70, 8.66	0.304	2.19	-2.11, 6.50	0.318

In addition, a regression analysis comparing the mean difference (difference between the mean score at Day 0 and at Day 60) across various age groups and professions was also conducted (Table 2). Across the various age groups; the retention was significantly lower in the 45-54 age group compared to the 18-24 age group, both at the univariate (MD: 4.98; 95% CI: 2.02 to 7.94) and multivariate levels (MD: 5.14; 95% CI: 2.15 to 8.13). Retention was similar between all other age groups compared to the 18-24 years age group both at the univariate and multivariate levels. The mean difference was significant only for the 45-54 age group. Across the various professions, there was no difference in retention between any of the professions at the univariate or multivariate level. Table 2 depicts the adjusted and unadjusted mean differences.

**Table 2:** Mean differences with unadjusted and adjusted covariates

Mean Differences With Unadjusted and Adjusted Covariates							
	Mean Difference (0 Day – 60 Day)	Unadjusted			Adjusted		
		Coef.	95% CI	p-value	Coef.	95% CI	p-value
Age Group							
18-24	19.51		Ref.			Ref.	
25-34	20.60	1.09	-0.96, 3.15	0.296	1.2	-0.90, 3.29	0.263
35-44	20.50	1	-1.09, 3.08	0.348	1.05	-1.08, 3.17	0.334
45-54	24.49	4.98	2.02, 7.94	0.001*	5.14	2.15, 8.13	0.001*
Profession							
Emergency Nurse	22.00		Ref.			Ref.	
Military Officer	20.23	-1.77	-10.91, 7.38	0.705	-2	-11.07, 7.06	0.665
Military Soldier	20.66	-1.34	-10.21, 7.54	0.768	-1.49	-10.29, 7.32	0.741
Other	21.86	-0.14	-9.43, 9.15	0.977	-0.08	-9.29, 9.14	0.987
Paramedic	20.20	-1.8	-12.98, 9.38	0.752	-1.52	-12.62, 9.58	0.788
Practical Nurse	19.00	-3	-20.68, 14.68	0.739	-3.05	-20.57, 14.47	0.733
Professional Role unknown	20.13	-1.88	-12.24, 8.49	0.723	-1.83	-12.11, 8.45	0.727

## Discussion

The aim of this study was: 1) to assess the level of competence of UAE military personnel in the use of sieve triage using a pre and post-course questionnaire; 2. to assess the knowledge retention at Day 0, Day 30, and Day 60 post-delivery of the course.

A sample size of 456 was obtained from six consecutive cohorts tested after delivery of training. One hundred percent compliance was obtained at the retesting date as per the requirements of the military education pre-deployment, which ensured an optimal response rate. The participants' age ranged from 18-54, out of which 80% were soldiers with no previous medical background and basic educational background. Nine percent of the respondents were officers and the rest included nurses and paramedics. Nine percent, however, were respondents who did not identify themselves with any specific professional groups.

The results from the study illustrated that the combined mean scores for the cohort were 96.81 at Day 0, 87.37 at Day 30; and 76.1 at Day 60. This showed a regression in knowledge retention by 20.71 over a 60 day period with the mean scores being highest at Day 0 and lowest at Day 60. This is consistent with several similar studies looking at knowledge retention post-delivery of training courses where a clear reduction of knowledge is noted during interval testing [9]. The regression analysis conducted suggested that at the univariate level, the mean scores reduced significantly at Day 30 and at day 60 and remained significant at Day 30 and Day 60 compared to Day 0 when adjusted for age and profession [10-11].

As regards knowledge enhancement as the effect of the training, at baseline, it was found that immediately post-course delivery, the combined mean scores across all cohorts was 96.81, which showed the effectiveness of the course material and the medium of course delivery to predominantly non-medical personnel [12]. In this study, knowledge increase was found immediately after the training, but it was not retained after 30 and 60 days, respectively, and therefore knowledge retention was not maintained [13]. It is suggested that short training led to increased knowledge but to be able to retain this knowledge, there is a need to conduct regular and periodic refreshers, especially for such incidents that are infrequent and the chance to use this knowledge practically is limited [14].

During the analysis of the data, mean scores were compared across the various age groups, which participated in the course. When the overall mean scores were compared across the age groups, the mean scores were significantly higher for the 25-34 and 35-44 age groups compared to the 18-24 age group, both at the univariate and at the multivariate level. However, the overall mean scores did not significantly differ for the 45-54 age group compared to the 18-24 age group both at the univariate and multivariate levels. Knowledge retention was, therefore, higher in the age group between 25 and 44, which is not in line with similar studies looking at knowledge retention across age groups, and further studies looking specifically at age variations and retention of knowledge will need to be done over a longer study period [15].

We also conducted a regression analysis comparing the mean difference (difference between the mean score at Day 0 and at Day 60) across various age groups and professions. Across the various age groups, retention was noted to be significantly lower in the 45-54 age group compared to the 18-24 age group, both at the univariate and multivariate levels. Retention was also similar between all other age groups compared to the 18-24 years age group both at the univariate and multivariate levels, and the mean difference was significant only for the 45-54 age group. In addition, across various professions, there was no difference in retention between any of the professions at the univariate or multivariate level.

It was assumed that there would be a significant difference when the overall mean scores were compared across the professions due to differences in the level of basic education; however, when the mean scores were collated, they did not statistically differ for any of the professions when compared to the military officer at the univariate level [16]. At the multivariate level; the overall mean scores were significantly lower for the ‘other’ profession compared to the military officer. Further on, there was no difference in the overall mean scores for any of the other professions compared to military officers at the multivariate level [17]. The lack of significant differences between the various professionals in retention of knowledge and skill suggests that practice with feedback and not professional training would have the greatest effect on retention [9-16].

A cohort study done in Mozambique showed that in six months after the assimilation of knowledge, there was a deficit in knowledge retention and resulting in loss of information [18]. There were several reasons identified, which are similar to cohorts in the current study. These included a decrease in the performance interfering in perceptual mechanisms and levels of attention, the willingness of the subjects to learn, the logic of the presented content, and the role of the trainers to make the content interesting to learn in having new concepts. Another suggestion to assist in its retention is relevance demonstrated to the existing situation, especially as the practical use of such a tool is practically difficult due to the low probabilities of such events happening [16-17].

The study had some limitations that the researchers tried to address during the course of delivery. First, the test questions were non-verbal and identical to all cohorts, which prevented researcher bias in subjects answering questions verbally and preventing conflict. Second, the research questionnaire was delivered to an Arabic-speaking population and the questions were translated into Arabic, thereby minimizing a language bias. Third, the survey group was randomly selected to reduce selection bias and six consecutive cohorts were tested randomly without any pre-selection. Fourth, situational bias was minimized due to the survey being conducted concurrently and prospectively. Last, the effect of non-responders was minimal, and 100% compliance was maintained due to military regulation of training cohorts to be re-tested as a mandatory requirement prior to deployment. However, one bias could not be rectified due to a cohort of respondents who did not identify with any of the professional groups and accounted for 9% of the total cohorts.

## Conclusions

In conclusion, knowledge retention from the delivery of sieve triage training among UAE military personnel decreased after 60 days, and therefore, there is a need to conduct regular and periodic refreshers, especially to topics that are not applied regularly. This could be in the form of more frequent training and continued medical education either through face-to-face learning or e-learning.

## Figures and Tables

**Figure 7:**
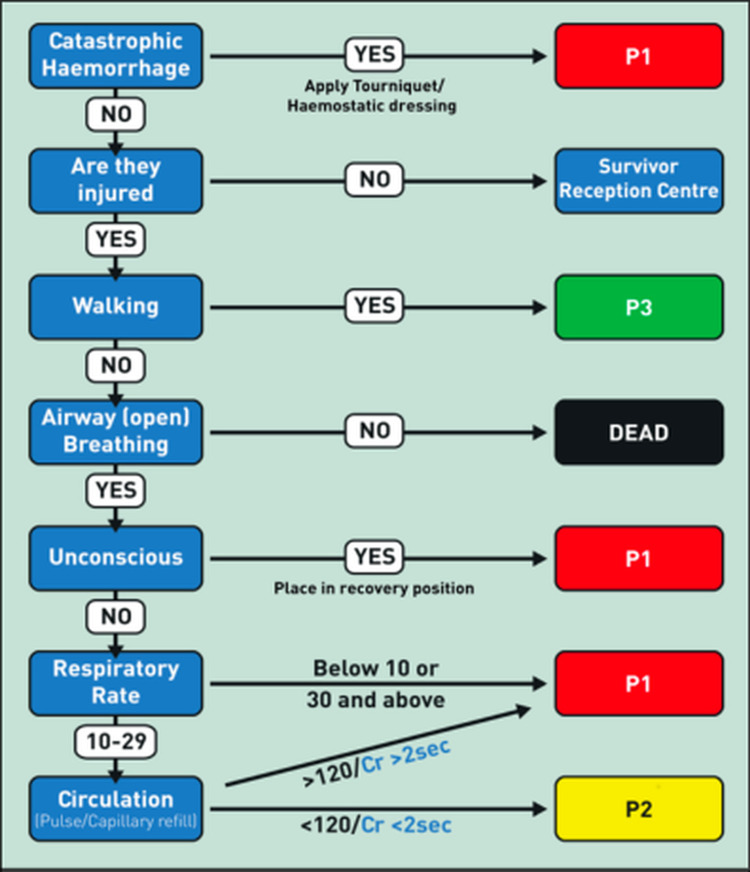
Triage algorithm

## Appendices

Examples of questions used in the survey

·       You are the first responder in a Mass Casualty Incident which has involved an IED explosion and after initial triage you are managing a patient who has an above knee amputation with a weak and thready radial pulse of 130. What is your best initial option for treatment? 

A.      IV fluid bolus

B.      Tourniquet

C.      Direct pressure

D.     Leg lift

E.      Celox

·       You are the first responder in Mass Casualty Incident which has involved a truck which has rolled over into a ditch and you have 5 victims. Please place each patient into an appropriate triage category based on your initial assessment. 

A.      30-year-old male who has self-extricated and walking on the scene 

B.      37-year-old male who is lying supine and on initial assessment is not breathing with no response with a simple airway maneuver 

C.      42-year-old male who is lying in a ditch but breathing spontaneously with a respiratory rate if 27 and a pulse of 123

D.     39-year-old patient sitting on the edge of the road is unable to walk suffering from abdominal pain. Respiratory rate 23 and pulse rate 110

E.      33-year-old who is lying on the road unable to mobilize and calling for help. At the first assessment the respiratory rate was 33

I.         Green; Yellow; Red; Black

·       You are the first responder in a mass casualty incident involving a rocket attack on a facility which has left 20 people dead and you are responsible for treating five casualties who are still on the scene. Please choose the best triage category for each.

A.     30-year-old male who is unconscious with no respiratory effort but on airway manipulation starts breathing

B.      33-year-old who is lying among the bushes complaining of severe lower limb pain with a pool of blood visible. He is respiratory rate is 36 and pulse 122

C.      37-year-old male who walks around the scene but appears confused

D.     42-year-old male who is not breathing and on opening the airway there is no response 

E.      39-year-old male who is walking and complains of shortness of breath with a respiratory rate of 18 and pulse of 110 

I: Green; Yellow; Red; Black
